# The Prevention and Immediate Treatment of Lacerated Cervix

**Published:** 1887-03

**Authors:** C. C. Frederick

**Affiliations:** Assistant in Obstetrics in Niagara Medical College


### *THE PREVENTION AND IMMEDIATE TREATMENT OF LACERATED CERVIX.**

By C. C. FREDERICK, M. D.,

Assistant in Obstetrics in Niagara Medical College.

Lacerated cervix uteri, with all the ills that its existence may inflict upon the woman, and the operations for the repair of the same, have been the subject of hundreds of pages, both in the standard and periodical literature of medicine. Much, also, has been written about the repair of lacerated perineum and its prevention. There are the methods recommended by Hohl, Olshausen, Ahlfeld, Goodell, Fasbender, Lusk, and others. But there is comparatively little written concerning the prevention of lacerated cervix. The investigations of the gynæcologists, in recent years, have brought to light the errors of the obstetrician, both of omission and commission.

We have learned that lacerations of the cervix and perineum are frequent—much more so than was ever dreamed of. We have been taught strictly to guard against the laceration of the perineum, and I believe it to be the almost universal practice to-day to look to its prevention, and, in case of its occurrence, to close the rent by suture. I have heard physicians, whose labor cases have run up into the hundreds, avow that they never

*Read before Buffalo Medical Club, Nov. 10, 1886.

had a lacerated perineum. Why? Because they never looked for it. I also have heard the same say that they never had a lacerated cervix. Why? Because they never looked for it. I believe that the majority of the profession, who now give so much attention to the perineum and its repair, seldom or never look for laceration of the cervix, or seek to prevent it. I think I am safe in making the statement that a deep laceration of the cervix is a more dangerous lesion for the puerperal period, and may be far more detrimental to the subsequent health of the woman, than a lacerated perineum possibly can be.

If this statement be true, why should not the accoucheur give as much care to the cervix as to the perineum, or even more?

What are the most common causes of lacerated cervix:

(1) Early rupture of the membranes.

(2) Tedious first stage, ordinarily known as rigid os.

(3) Precipitate labor, with powerful pains.

(4) Forcible dilatation to hasten labor, as in puerperal eclampsia.

(5) High forceps operations.

(6) Manual delivery by version.

(7) Breech labors, with rapid extraction of the after-coming head.

(8) Ergot.

(9) In cases where the anterior lip of the cervix is caught between the head of the child and the pubic bone, and prevented from retracting simultaneously with the posterior lip.

Early rupture of the membranes more commonly occurs in breech or abnormal presentations of the child, and in these laceration is most liable to occur during the delivery of the after-coming head, of which I shall speak further on. Early rupture in vertex presentations, leaving the head to act as the dilating body, tends to excite expulsive efforts before complete dilatation, and before the organic changes have taken place, necessary to dilatation. These too early expulsive pains, therefore, are liable to force the head through the partly-opened cervix, and all that will not stretch must tear. How may we prevent this. Give chloroform—not to complete anæsthesia, but enough to quiet the reflex irritability—to favor relaxation, and then, between pains, push the vertex up away from the cervical ring, as naturally occurs in the interim of normal first-stage pains. Or, if the premature rupture be very early, and the cervix is but slightly dilated, and pains become hard or continuous, accompanied by a rigid os, administer morphine hypodermically, and give chloroform till the morphia begins to act; or give chloral, per rectum. If these fail to accomplish our purpose, Barnes' bags may be employed. If these are of no service, as they often are not, apply forceps, if the cervix be open enough, and then dilate by intermittent traction, giving a little chloroform to favor relaxation.

I believe that most of us, in our anxiety to save time and get our patients into the second stage of labor, are prone to rupture the membranes sometimes before the cervix is fully dilated. I know it to be the practice of some to do this, if the cervix be soft and thin, long before it is open enough to allow the head to pass without tearing. We must admit, however, that in some cases of slow first-stage, with thick cervix and great pain, the membranes being intact, that no better treatment can be instituted than to rupture the membranes, thus allowing the head to come down into the cervical ring, and help to dilate it. Tedious first-stage is to be treated about as indicated for early rupture of the membranes. In these, anodynes act more uniformly, and the Barnes dilators stretch the cervix better, than when the membranes are ruptured.

Precipitate labor, with powerful pains, the soft parts being dilatable and readily dilating, is not abnormal. But precipitate labor, due to too powerful pains, forcing the child through resisting tissues, is pathological, and the pains must be promptly controlled by chloroform to complete anæsthesia, or, if more enduring effect is desired, morphia, hypodermically administered. Forcible dilatation, at all times, is prone to produce rupture.

In puerperal eclampsia, I believe manual dilatation, or dilatation by Barnes' bags, while holding the patient under morphia or chloroform, may be accomplished sufficiently to prevent deep lacerations. From the reported cases, I am inclined to believe in the efficacy of veratrum viride, in large doses, to produce relaxation and rapid dilatation in these cases. As for placenta prævia, and other conditions in which version, manual or high instrumental delivery is necessary, the cervix may be well dilated, if we only exercise patience and work systematically to get it, before proceeding to deliver.

Deep lacerations, extending above the vaginal junction, are most often produced in breech deliveries or podalic version, when the head is extracted by force through an imperfectly dilated os. The remedy, therefore, is full dilatation, to extract the head as gently as possible, and in extreme flexion. And extreme flexion is best secured by firm supra-pubic pressure, and a finger in the child's mouth.

Ergot—what shall I say of it. I am almost ashamed to assume that anyone would use it in doses sufficiently large, during the first stage of labor, to have it classed as one of the causes of lacerated cervix.

When the anterior lip is incarcerated between the descending occiput and the pubic walls, and prevented from retracting simultaneously with the posterior lip, push the head back between pains, and with the ends of two fingers introduced, slip the lip up over the occiput.

Having completed the labor, I believe it the duty of the physician to examine his patient systematically for all lesions of continuity. The perineum can be seen, but the cervix cannot be so readily. Slight lacerations of the cervix are difficult to feel, but deep ones are not. By examining every cervix after labor, one may become quite expert in feeling these tears. First wash out the vagina with a hot sublimated douche, after securing firm uterine, contraction and retraction. With the cervix clean, the edges may readily be felt. Lacerations which do not extend near to the vaginal junction generally heal rapidly during involution, providing that septic infection of contiguous connective tissues does not occur. I have several times proven this in my own cases by examination a month or more after labor.

I have seen two cases of lacerated cervix, in which hemorrhage occurred from the tear, one of them severe, to the point of syncope. The milder of the two occurred in my own practice. Both were primiparæ. With the completion of the third stage and firm contraction and retraction of the uterus, the blood began to gush from the patients, and in both I stopped the flow by passing my hand into the vagina and using my first and second finger as a clamp, at the same time giving the patient as hot a vaginal douche as my hand would bear.

In my own case, after temporarily stopping the hemorrhage, I placed the woman in Sims' position, put in a Sims speculum, and drawing the uterus well down with a tentaculum, and by maintained supra-pubic pressure, I sewed up the laceration with three silver wire sutures. It is surprising to see how near to the vaginal outlet the uterus can be pushed by supra-pubic pressure and how readily the stitches can be passed. The only obstacle is the continuous oozing of blood, and this may be, to a great extent, prevented by first securing firm retraction of the uterus and by giving a hot vaginal douche just before turning the patient on the side.

I removed the stitches in about fifteen days, to find the laceration healed.

The physician in attendance upon the second case did not favor repairing the tear. I am interested to know if it ever healed.

The plan of suturing the torn cervix was first successfully tried, I believe, by Prof. Montrose A. Pallen. The only condition for which it is recommended is the occurrence of hemorrhage. I do not see why a deep laceration down to the vaginal junction, even if there be no serious hemorrhage, should not be sewed.

